# The Cerebral Protective Effect of Novel Erinacines from *Hericium erinaceus* Mycelium on In Vivo Mild Traumatic Brain Injury Animal Model and Primary Mixed Glial Cells via Nrf2-Dependent Pathways

**DOI:** 10.3390/antiox13030371

**Published:** 2024-03-19

**Authors:** Kam-Fai Lee, Yung-Yu Hsieh, Shui-Yi Tung, Chih-Chuan Teng, Kung-Chuan Cheng, Meng-Chiao Hsieh, Cheng-Yi Huang, Ko-Chao Lee, Li-Ya Lee, Wan-Ping Chen, Chin-Chu Chen, Hsing-Chun Kuo

**Affiliations:** 1 Department of Pathology, Chang Gung Memorial Hospital, Chiayi 613016, Taiwan; lkf2002@cgmh.org.tw; 2 Division of Gastroenterology and Hepatology, Department of Internal Medicine, Chang Gung Memorial Hospital, Chiayi 613016, Taiwan; ivan@cgmh.org.tw (Y.-Y.H.); ma1898@cgmh.org.tw (S.-Y.T.); 3 College of Medicine, Chang Gung University, Taoyuan 333323, Taiwan; 4 Department of Nursing, Division of Basic Medical Sciences, Chang Gung University of Science and Technology, Chiayi 613016, Taiwan; ccteng@mail.cgust.edu.tw; 5 Research Fellow, Chang Gung Memorial Hospital, Chiayi 613016, Taiwan; 6 Division of Colorectal Surgery, Department of Surgery, Chang Gung Memorial Hospital-Kaohsiung Medical Center, Kaohsiung 833401, Taiwan; topguncheng@cgmh.org.tw (K.-C.C.); kmch4329@gmail.com (K.-C.L.); 7 Division of Colon and Rectal Surgery, Department of Surgery, Chang Gung Memorial Hospital, Chiayi 613016, Taiwan; chris0912@cgmh.org.tw (M.-C.H.); bluesky@cgmh.org.tw (C.-Y.H.); 8 College of Medicine, Chang Gung University, Kaohsiung 833401, Taiwan; 9 Biotech Research Institute, Grape King Bio Ltd., Taoyuan 325002, Taiwan; ly.lee@grapeking.com.tw (L.-Y.L.); wp.chen@grapeking.com.tw (W.-P.C.); gkbioeng@grapeking.com.tw (C.-C.C.); 10 Research Center for Food and Cosmetic Safety, College of Human Ecology, Chang Gung University of Science and Technology, Taoyuan 333324, Taiwan; 11 Chronic Diseases and Health Promotion Research Center, Chang Gung University of Science and Technology, Chiayi 613016, Taiwan

**Keywords:** *Hericium erinaceus* mycelium, erinacine C, mild traumatic brain injury, Nrf2-mediated antioxidant pathway

## Abstract

*Hericium erinaceus*, a consumable mushroom, has shown a potential to enhance the production of neuroprotective bioactive metabolites. Traumatic brain injury (TBI) often leads to cognitive, physical, and psychosocial impairments, resulting in neuroinflammation and the loss of cortical neurons. In this research, the effects of *H. erinaceus* mycelium, its derivative erinacine C, along with the underlying mechanisms, were examined in terms of oxidative stress modulation and neurological improvement in a rat model of mild traumatic brain injury (mTBI). Male Sprague-Dawley rats were administered diets containing *H. erinaceus* mycelium and erinacine C following experimental brain injury; these supplements were continued throughout the recovery phase. The binding activity of NF-E2-related factor 2 (Nrf2) near antioxidant genes in mixed glial cells was measured by chromatin immunoprecipitation-quantitative polymerase chain reaction (ChIP-qPCR). The motor beam walking test revealed that dietary supplementation of *H. erinaceus* mycelium resulted in modest improvements in spatial memory while inhibiting neuron cell death and microglial activation according to brain histological examination. These findings were further corroborated by the upregulation of several antioxidant enzymes (catalase, glutathione reductase, thioredoxin reductase, and superoxide dismutase) and phospho-CAMP-response element-binding (p-CREB) levels in the mTBI model treated with *H. erinaceus* mycelium. Erinacine C treatment led to significantly reduced brain inflammation and normalization of mTBI-induced deficits through the modulation of the Nrf2 activation pathway and upregulated expression of numerous Nrf2-binding antioxidant genes such as catalase, thioredoxin reductase, superoxide dismutase, and brain-derived neurotrophic factor. This study demonstrates the potential of *H. erinaceus* mycelium and erinacine C in facilitating recovery following mTBI, including the prevention of neuronal injury and inactivation of microglia through the Nrf2-mediated antioxidant pathway in vivo.

## 1. Introduction

Traumatic brain injury (TBI) may have long-term consequences, including anxiety, depression, paralysis, and memory impairment [1]. Patients with TBI often experience cognitive decline and short-term memory loss and require prolonged rehabilitation [2]. Thus, the development of effective drugs for TBI treatment is crucial [3]. Mild traumatic brain injury (mTBI) is the most reported type of TBI worldwide, with approximately 1.7 million annual cases [4]. It frequently occurs in contact sports such as boxing, hockey, soccer, and American football, as well as in victims of child abuse and military personnel [5]. mTBI, a form of closed head injury, can lead to cumulative and long-term behavioral symptoms, neuropathological changes, and neurodegeneration [6,7]. mTBI can result in cerebral edema, characterized by the accumulation of excess fluid in the brain, influencing both neurons and microglia and impacting both the structural and functional aspects of the brain. The edema induces an increase in intracranial pressure, subsequently reducing cerebral blood flow. Neurons, highly sensitive to alterations in blood flow and pressure, may experience dysfunction and damage due to prolonged pressure elevation. Moreover, cerebral edema initiates an inflammatory response, activating microglia—the resident immune cells of the central nervous system. These activated microglia release pro-inflammatory cytokines and reactive oxygen species (ROS), contributing to the phagocytosis of cellular debris and damaged neurons. To simulate the clinical consequences of mTBI, various models, such as the modified weight-drop technique, have been developed [8]. This technique involves applying a glancing impact to the head of a freely moving rodent, resulting in acceleration, deceleration, and rotational forces on the brain; the process does not require scalp incision or skull helmets [9,10]. Our animal model replicates clinically relevant biomechanics of the pathophysiology of the injury and microglial activation, with a low mortality rate, making it suitable for screening new therapies for mild concussive injuries and a wide range of behavioral and molecular studies [11,12,13]. Therefore, developing more effective drugs using natural compounds derived from edible mushrooms is crucial to prevent neuronal death and microglia activation in a reliable model of mTBI [14]. In TBI, the primary injury, caused by an external force, causes mechanical tissue deformation, resulting in diffuse neuronal depolarization and the release of excitatory neurotransmitters, leading to a massive influx of calcium [15]. Calcium sequestration in the mitochondria disrupts calcium homeostasis, leading to energy deficits, free radical formation, and apoptotic initiation [16]. Neurotoxic byproducts of lipid peroxidation have gained interest in various neurological diseases associated with oxidative stress; these byproducts contribute to oxidative damage in TBI [17]. Oxidative neurodegeneration plays a significant role in aggravating morphological responses and impairments in behavioral recovery, resulting in functional deficits. Enzymatic antioxidants, such as catalase (CAT), glutathione peroxidase, and superoxide dismutase (SOD), serve as major defense enzymes against superoxide radicals, protecting the brain from the damage due to reactive oxygen species (ROS) [18,19,20,21]. Considering the role of oxidative stress in promoting neuronal degeneration after mTBI, this study aimed to investigate the neuroprotective effects and functions of *Hericium erinaceus,* which can provide insights into antioxidant treatment post-TBI.

*H. erinaceus*, commonly known as Lion’s Mane or Yamabushitake, is a mushroom consumed as food in Japan and China [22]. This mushroom has therapeutic properties, which include antioxidant, hypolipidemic, hemagglutinating, antimicrobial, anti-tumorigenic, and endoplasmic reticulum stress-modulated activities [23]. Extracts from the fruiting body and mycelium of *H. erinaceus* provide various health benefits and activate physiological functions [24]. The main active agents in the cultured mycelia of *H. erinaceus* are erinacines A-I, the diterpenoid compounds known to stimulate the synthesis of nerve growth factor (NGF) in astrocytes, leading to a range of physiological effects [25] of particular interest is erinacine C, an important secondary metabolite produced by *H. erinaceus* mycelium. Erinacine C can promote the synthesis of NGF by astrocytes in rodents more effectively compared to epinephrine, a previously known NGF stimulant, suggesting that erinacine C can be potentially used as a treatment for neurodegenerative diseases. Oral intake of *H. erinaceus* mycelium can protect against ischemic brain injury and 1-methyl-4-phenyl-1,2,3,6-tetrahydropyridine (MPTP)-induced neurotoxicity by inhibiting ER-stress-mediated cell apoptosis in vivo [26]. However, whether erinacine C is present in *H. erinaceus* mycelium and its neuroprotective activity against ischemic brain injury remains poorly understood. Erinacine C administration after mTBI could prevent neuron injury by inactivating microglia and increasing the expression of several antioxidant enzymes in the NF-E2-related factor 2 (Nrf2) activation pathway.

## 2. Materials and Methods

### 2.1. Hericium Erinaceus Extracts and Analysis of Erinacine C

To extract erinacine C from the fresh mycelium of *H. erinaceus*, they were refluxed with ethanol. The resulting ethanol solution was concentrated using a vacuum, which gave a brown extract that was partitioned with H_2_O/EtOAc (1:1) to produce two layers—a H_2_O layer and an EtOAc layer, according to the previous study [27]. The EtOAc layer was then concentrated and fractionated to chromatography on a silica gel column to obtain seven fractions. Fraction VI, which was the eluate of n-hexane/EtOAc (1:2), was further separated on Sub-fraction VI-1 and was then purified to ultimately obtain erinacine C. The several different batches of mycelium extract were further purified to isolate erinacine C, investigated by the therapeutic potential of erinacine C and the effect of oral administration of *H. erinaceus* mycelium in a weight-drop-induced model of mTBI.

**Figure 1:**
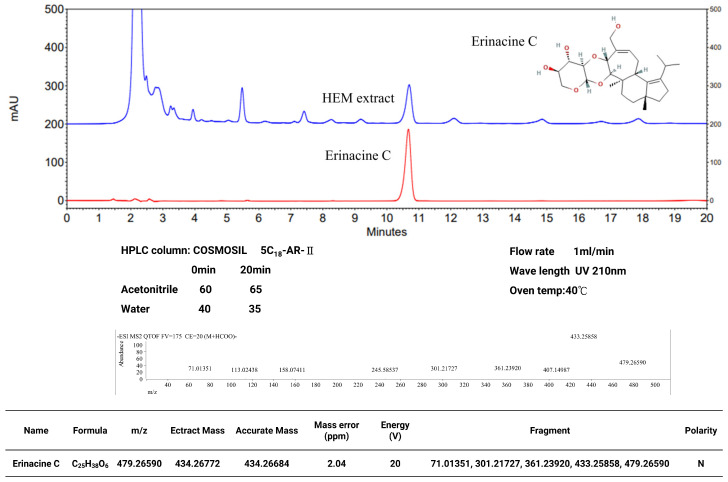
High-performance liquid chromatography (HPLC) analysis of erinacine C. HPLC analysis and liquid chromatography-mass spectrometry analysis of the ethanol *Hericium erinaceus* mycelium (HEM) extract. The retention time peak at 10.5 min represents erinacine C (observed through UV detection at 340 nm). LC-QTOF/MS spectra of Erinacine C with parent ion detected at m/z 479.26590.

HPLC analysis of erinacine C was conducted following a previously established method with slight modifications [27]. The COSMOSIL 5C_18_-AR-II analytical column was utilized for separation at a temperature of 40 °C. The mobile phase consisted of two solvents, namely ACN (A) and H_2_O (B), and two different gradients were employed. The gradient elution protocol was as follows: 0–20 min, 60–65% (A). Erinacine C was detected at a retention time of approximately 10.5 min, with a flow rate of 1.0 mL/min, and monitored using a UV wavelength of 210 nm. The presence and quantity of erinacine C in the *H. erinaceus* extract obtained using 85% ethanol were confirmed and quantified by HPLC analysis, as depicted in Figure 1.

### 2.2. Induction mTBI Brain Injury and Drug Administration

Adult male Sprague-Dawley rats, aged four to five weeks and weighing approximately 120 ± 20 g, were individually housed in cages with a 12-h light/dark cycle, providing free access to water and food. The Institutional Animal Care and Use Committee of Chang Gung University of Science and Technology approved all animal care procedures and general protocols for animal use (IACUC Approval: 2018042501). The animal model of cerebral trauma was built according to Mychasiuk et al. [12,28,29]. The bottom module of the animal impact model was constructed. First, tin foil was pasted onto a U-shaped platform made of a transparent plastic rectangular box (38 × 27 × 27 cm^3^), which contained a soft sponge (38 × 25 × 15 cm^3^) as recoil. The pipe with the specification of 2.2 cm (diameter) ×1.5 m was adjusted. It was fixed by support and placed 3.5 cm above the tin foil, where a laboratory rat’s head was located. Afterward, a weight connected to a metal ring was attached to a fishing line, allowing it to fall freely onto the laboratory rat’s head. A 150 g weight at a height of 50 cm was used as a free-falling body for impact. An intramuscular injection (1 mg/kg) of the anesthetic 50 mg/kg of Zoletil 50 plus 23 mg/kg of Xylazine (Ropum) was applied to the laboratory rat. The rat was completely narcotized before the operation. The rat’s body temperature was maintained by an animal incubator during the experimental process. Five groups (six rats in each group) were randomly assigned to a sham control (CL) group, a mTBI animal model group, and *H. erinaceus* wet mycelia groups (108.5 mg/kg and 217 mg/kg), and erinacine C groups (2 mg/kg). Related animal behavior patterns were performed on Day 1 (after TBI) and Day 2 to 6 (after treatment) to observe whether the rats’ conditions were improved by the erinacines. After the rats’ brain tissues were collected, pathological sections, immunostaining, cerebral cortex, subcortex, and hippocampus were used to analyze the growth of neural cells and the mechanism for inhibiting the inflammation of microglia on Day 7.

### 2.3. Verification of mTBI with the Beam Walking Test

A wide range of rodent motor tests are available for assessing deficits in animal models. Among these, the beam walking test is commonly employed to evaluate rodent gait under conditions that challenge their balance. In this test, animals are required to traverse an elevated beam with a narrow diameter, as described in previous studies [30].

### 2.4. Reagents and Antibodies

Mouse monoclonal antibodies against NeuN, iba1, β-actin, catalase, thioredoxin, superoxide dismutase, and BDNF, and rabbit poly monoclonal antibodies against Nrf2 were purchased from Santa Cruz Biotechnology (Santa Cruz, CA, USA). Rabbit monoclonal antibodies against phospho-CREB were purchased from Cell Signaling Technology (Beverly, MA, USA). LPS (*E. coli* O111:B4, protein contaminants ≤ 2.0%, nucleic acid contaminants ≤ 2.5%) was purchased from EMD Chemicals, Inc. (Darmstadt, Germany).

### 2.5. Preparation of Primary Mixed Glia Cultures and Cell Treatment

Primary mixed glia cultures were prepared from the whole brains of postnatal day-1 C57BL/6J mouse pups (*n* = 10), following previously established protocols [31]. The mixed glia cultures consisted of approximately 80% astrocytes and 20% microglia, as confirmed by immunostaining with cell-type specific markers, including Neu-N for microglia, Iba-1 for microglia, and GFAP for astrocytes. After replacing the culture media and allowing an additional 6-h incubation, the cells were treated with LPS. Immunostaining with specific antibodies allowed for the visual counting of cells expressing the respective markers, microglia (Neu-N), microglia (Iba-1), and astrocytes (GFAP).

### 2.6. Immunohistochemistry

Immunohistochemistry (IHC) staining was conducted using the Vectastain Universal Elite ABC Kit (Bio-Rad, Hercules, CA, USA), employing a biotinylated secondary antibody. Negative controls were generated by omitting the primary antibodies. For scoring, the presence of brown-stained cytoplasm was considered a positive signal, and three slides were used for evaluation. The expression levels of iba1, NeuN, catalase, glutathione, thioredoxin, and superoxide Dismutase 1 were quantitatively assessed using a microscope (Olympus, Tokyo, Japan) equipped with the medical image analysis system (Image-Pro Plus 6.0). Histochemical analysis was performed to quantitatively evaluate the expression proteins, following a previously described protocol [32].

### 2.7. Histopathological Evaluation

Brain tissue samples were fixed in buffered formalin, embedded in paraffin, and sectioned into 4 μm-thick slides, transversely from the region of neuronal impairment, for subsequent hematoxylin and eosin (H&E) staining. Histopathological changes in neuronal cells (NeuN-positive) and microglia cells (iba1-positive) were evaluated using a light microscope (Olympus, Tokyo, Japan) at a high magnification of 200× for each slide. To obtain quantitative data, cortical and subcortical infarctions were blindly selected from each slide, and images were captured using an Image-pro Plus medical image analysis system. The assessment of cytoplasm staining was based on a positive score for brown staining in the slides. Protein expression levels were quantitatively analyzed using an Olympus Cx31 microscope equipped with an Image-pro Plus medical image analysis system. Digital images were obtained using a Canon A640 digital camera (Canon, Tokyo, Japan). Positive cell area and optical density were measured in three randomly selected microscopic fields (200× magnification) on each slide. The Immunohistochemistry (IHC) index was determined as the average integral optical density (AIOD), calculated as the positive area multiplied by the optical density, divided by the total area (AIOD = positive area × optical density/total area). The numbers of positive cells were recorded for further analysis, following established protocols [33].

### 2.8. Preparation of Total Cell Extracts and Immunoblotting Analysis

Cellular lysates were prepared by suspending brain tissue in a lysis buffer. The cells were subsequently disrupted by sonication, and the resulting lysates were subjected to extraction. The protein content in the supernatant was quantified using a suitable method. For immunoblotting, Immobilon-P membranes (Millipore, Bedford, MA, USA) were employed, along with the appropriate secondary antibodies. Signal detection was performed using an enhanced chemiluminescence Western blot kit (Bio-Rad, Hercules, CA), following established protocols [34].

### 2.9. Chromatin Immunoprecipitation (ChIP) Analysis

The cells were treated with 1% formaldehyde at room temperature to crosslink DNA and proteins, followed by treatment with glycine. Subsequently, cells were scraped using an SDS lysis buffer ((St. Louis, MO, USA). Immunoprecipitation was carried out by incubating the cells with specific antibodies against Nrf2, along with non-immunized rabbit IgG as a negative control, using a rotary shaker. For quantitative PCR, specific primers targeting the promoter region of the *BDNF*, *CAT*, *TrxR*, and *SOD1* genes were utilized, with their sequences provided in Table 1. The data were expressed as a percentage relative to a reference gene, as described previously. The reference gene was used to calculate the percent input for each experiment.

### 2.10. Statistical Analysis

All data were presented as mean ± SD and analyzed using either Student’s *t*-test or one-way analysis of variance (ANOVA) to compare between groups. A *p*-value of less than 0.05 was considered statistically significant [35]. The data were analyzed using the SAS software statistical package “SigmaPlot”, version 9.0 (SAS Institute, Cary, NC, USA).

## 3. Results

### 3.1. Hericium Erinaceus Mycelium Treatment Ameliorates Brain Damage in Rat Model with Mild Traumatic Brain Injury

**Figure 2:**
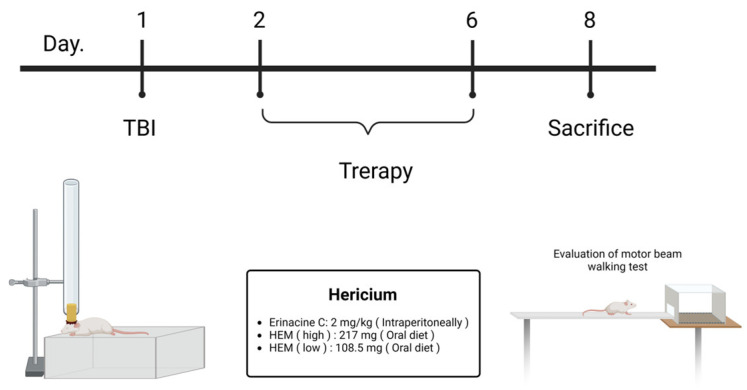
A simplistic flow chart of the therapeutic activities of *Hericium erinaceus* mycelium (HEM) in a model of mild traumatic brain injury (mTBI) for Sprague-Dawley rats. After the induction of mTBI, the rats exhibited significant motor dysfunction, as indicated by an increase in the time period on the beam walking test at days 2 to 6 by the mTBI apparatus. The rats were treated with HEM and its isolated erinacine C.

**Figure 3:**
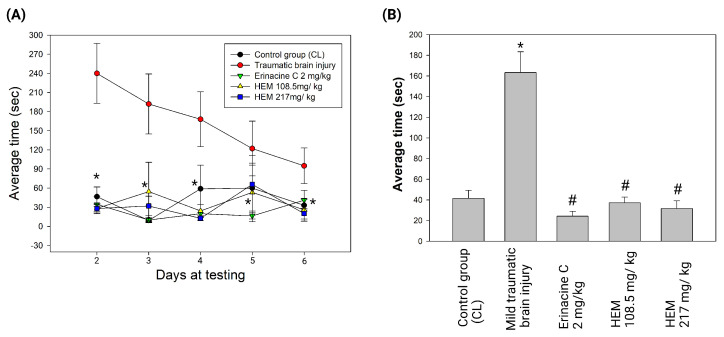
Graphical representation of the average differences between time-to-right of rats who experienced a single mild traumatic brain injury (mTBI) and *Hericium erinaceus* mycelium (HEM)-treated rats who experienced the beam walking test. (**A**) HEM and its isolated erinacine C-treated rats with mTBI exhibited a significant decrease in the duration needed to upright themselves from the supine position. * *p* < 0.05, as compared to the control group. (**B**) The average time of mTBI was quantitated and collected during testing for 6 days. * *p* < 0.05, as compared to the control group; # *p* < 0.05, as compared to the mTBI-induced group.

In this study, the fermentation process for *H. erinaceus* mycelium was established with minor modifications [27]. The freeze-dried powder of *H. erinaceus* mycelia was cultured. On the fermentation, ultrasonic oscillation was used to extract 0.5 kg of freeze-dried mycelia powder with 5 L of alcohol. The extract was then filtered and concentrated by decompression to obtain the alcoholic extract of *H. erinaceus* mycelium. The mycelium extract was further purified to isolate erinacine C, which has a PubChem CID of 10252378. In the high-performance liquid chromatography (HPLC) analysis with a flow rate of 1.0 mL/min and a scanning UV wavelength of 210 nm, erinacine C eluted at approximately 10.5 min retention time in the mobile phase (Figure 1). Next, the therapeutic potential of erinacine C and the effect of oral administration of *H. erinaceus* mycelium in protecting neurons in a weight-drop-induced model of mTBI were investigated. The experiment involved cerebral concussion induction on Day 1, followed by treatment with *H. erinaceus* mycelium and erinacine C on days 2 to 6. The behavior patterns of the rats were assessed using beam walking tests, and thus, the effects of *H. erinaceus* mycelium and erinacine C on mTBI-induced motor abnormalities and neuroinjury were evaluated (Figure 2). The rats were placed on an aluminum balance beam, and the number of limb falls and the time taken to walk across the beam were recorded. The mTBI group required more time to traverse the balance beam compared to the healthy control group. Moreover, the time needed to walk across the beam was significantly reduced, compared to the mTBI group, by approximately 1/4 to 1/3 after the administration of *H. erinaceus* mycelium orally and erinacine C intraperitoneally (Figure 3A). The total time taken to cross the beam over 5 consecutive days was also evaluated, and compared to the mTBI group, the *H. erinaceus* mycelium, and erinacine C treatment groups showed significantly reduced total times by 20%, 25%, and 22% (Figure 3B). These findings indicated that *H. erinaceus* mycelium and erinacine C mitigated the motor deficits induced by mTBI. On day 7, the rats were sacrificed, and their brain tissues were collected and subjected to immunohistochemistry for pathological evaluation.

### 3.2. Hericium Erinaceus Mycelium Prevents Neuron Cell Death and Alleviates Neuroinflammation in the Rat Model with Mild Traumatic Brain Injury

**Figure 4:**
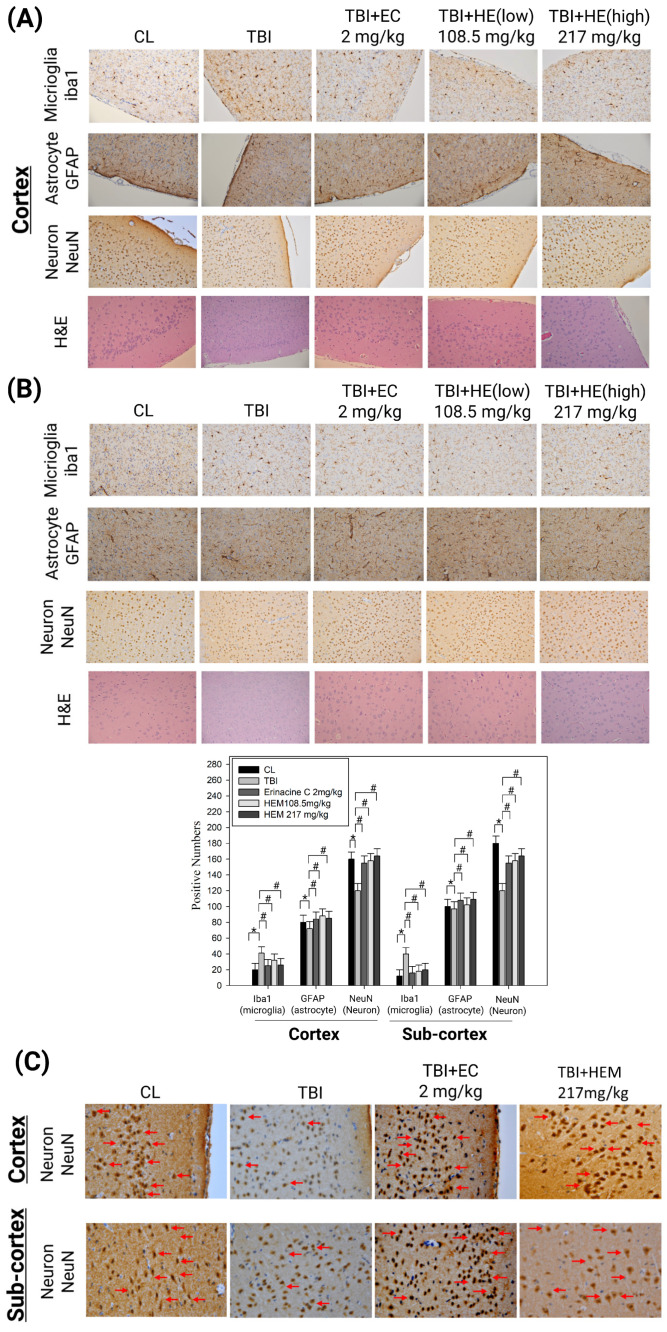
Effect of *Hericium erinaceus* mycelium (HEM) on brain histological examination in mild traumatic brain injury (mTBI) animal model. Rats were treated without (control group, CL) or with HEM by oral administration (108.5 and 217 mg/kg) and intraperitoneal erinacine C injection (2 mg/kg). Immunohistochemical staining of ionized calcium binding adaptor molecule 1 (Iba1) and NeuN of the brain revealed cortex (**A**) and subcortex (**B**) zones. Pathological examination and Iba1 and NeuN expression on brain histological examination in mTBI animal model were quantitative in brain tissue of cortex and subcortex zones. The cells were counted from 10 fields (200× magnification) of each brain sample. The results of statistical analysis are presented as the means of cells and were calculated per microscope field from six animals per group. * *p* < 0.05, as compared to the control group; # *p* < 0.05, as compared to the mTBI-treated group. Immunohistochemical staining of NeuN of the brain revealed cortex and subcortex zones (**C**). Pathological examination and NeuN expression on brain histological examination in the mTBI animal model were shown in morphological changes of neurons. The cells were imaged from 400× magnification of each brain sample (Arrowheads, axons, and dendrites).

In the mTBI rats group, we observed a decrease in cell counts, as indicated by the expression of the neuronal marker NeuN, in the cortex and subcortex of the cerebral region (Figure 4A,B, * < 0.05). However, in the rats treated with erinacine C and *H. erinaceus* mycelia through oral feeding (at doses of 108.5 mg/kg and 217 mg/kg), the number of normal neurons increased significantly in both the cerebral cortex and subcortex, at approximately 28%, 30% and 37% as well as 25%, 33%, and 38%, compared to the mTBI group for both cortical and subcortical structures (Figure 4A,B, # < 0.05). In rats subjected to mTBI, alterations in the expression of NeuN in the cortex and subcortex of the cerebral region can indicate morphological changes of neuronal loss and damage and repair function by quantitative analysis of NeuN staining intensity. The administration of *H. erinaceus* mycelium and erinacine C showed a more pronounced protective effect on the brain tissue in terms of preserving neuron counts and neuroplasticity or efforts to restore neuronal integrity in response to the treatment. Furthermore, in the mTBI rats group, the cortex and subcortical structure revealed an extended activation of microglia, indicated by the expression of ionized calcium binding adaptor molecule 1 (Iba1) (Figure 4A,B, * < 0.05). The activated microglia exhibited an inflamed and stellate appearance. However, the cerebral region of the rats receiving erinacine C intraperitoneally and *H. erinaceus* mycelium orally (at doses of 10.85 mg/kg and 217 mg/kg) showed a significant decrease in the number of activated microglia cells. The decrease in both the cerebral cortex and subcortex was approximately 62%, 75%, and 65%, as well as 50%, 65%, and 68%, compared to the number of microglia cells in the mTBI rats group (Figure 4A,B, # < 0.05). The anti-inflammatory response in the brain is not solely dependent on microglia activation; astrocytes also play a crucial role in modulating inflammation within the central nervous system (CNS) and can release anti-inflammatory molecules. However, the rats receiving erinacine C intraperitoneally and *H. erinaceus* mycelium orally (at doses of 10.85 mg/kg and 217 mg/kg) showed a mild increase in the number of activated astrocytes by both the cerebral cortex and subcortex was approximately 16%, 12% and 15% as well as 11%, 5% and 11%, compared to the mTBI rats group (Figure 4A,B, # < 0.05). This suggests that erinacine C and *H. erinaceus* mycelium treatment can attenuate microglial inflammation in the cerebral region. The administration of erinacine C and *H. erinaceus* mycelium extracts demonstrated a more pronounced protective effect on brain tissue, as evidenced by the preservation of neuron counts and the promotion of neuroplasticity, indicating efforts to restore neuronal integrity in response to the treatment. This is illustrated in Figure 4C, where elongated neuronal trees (arrowheads, axons, and dendrites) are observed arising from their cell bodies, compared to the cells in the mTBI-treated group (Figure 4C).

### 3.3. Post-Treatment with H. erinaceus Mycelium Reversed the Several Antioxidant Enzymes and Phospho-CAMP-Response Element-Binding Levels in a Rat Model of Mild Traumatic Brain Injury

**Figure 5:**
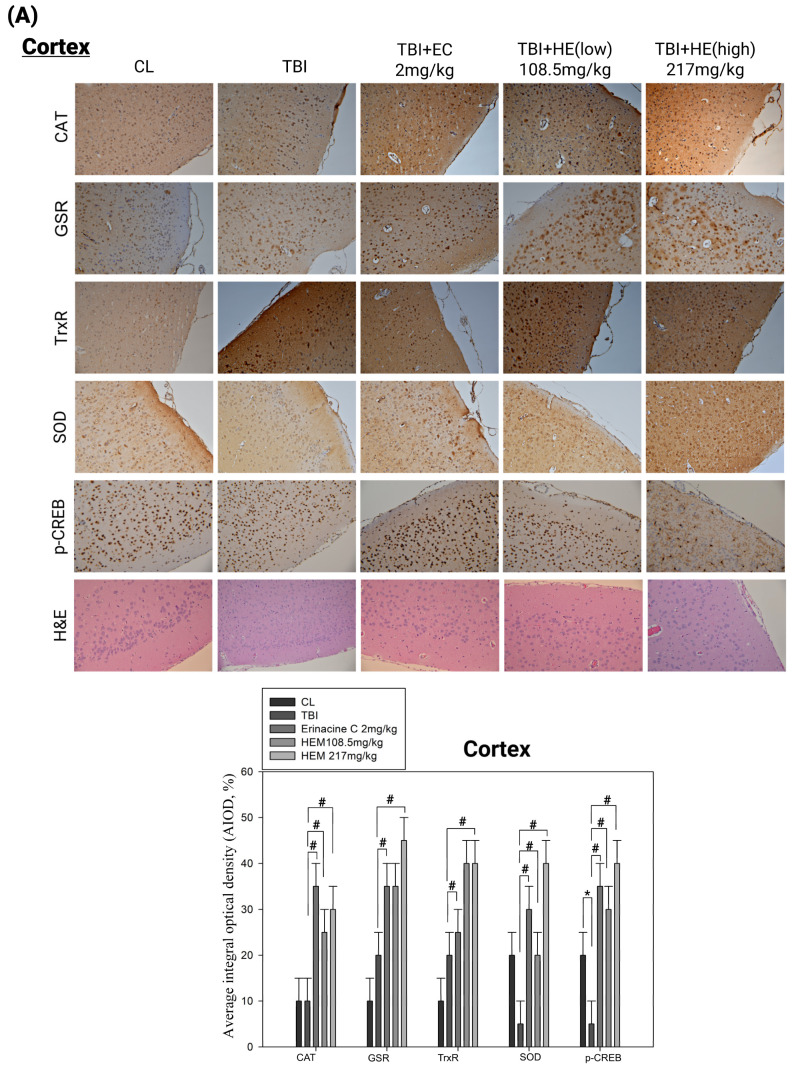

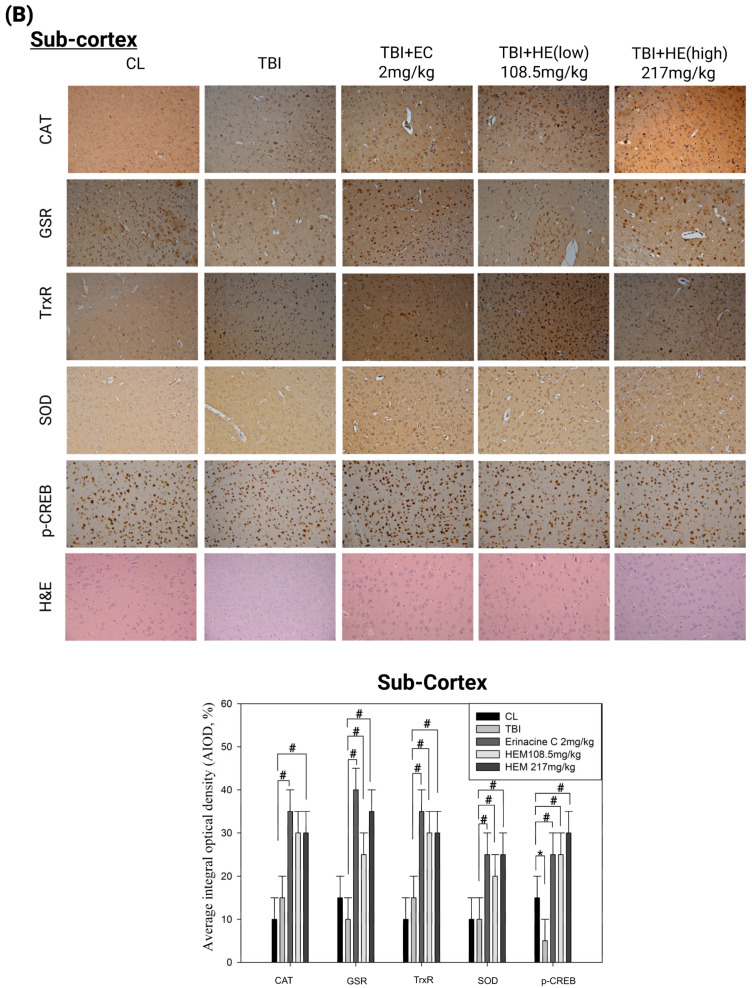
Effect of *Hericium erinaceus* mycelium (HEM) on brain histological examination in mild traumatic brain injury (mTBI) animal model. Rats were treated without (control group, CL) or with HEM by oral administration (108.5 and 217 mg/kg) and intraperitoneal erinacine C injection (2 mg/kg). Immunohistochemical staining of ionized calcium binding adaptor molecule 1 (Iba1) and NeuN of the brain revealed cortex (**A**) and subcortex (**B**) zones. Pathological examination and Iba1 and NeuN expression on brain histological examination in mTBI animal model were quantitative in brain tissue of cortex and subcortex zones. The cells were counted from 10 fields (200× magnification) of each brain sample. The results of statistical analysis are presented as the means of cells and were calculated per microscope field from six animals per group. * *p* < 0.05, as compared to the control group; # *p* < 0.05, as compared to the mTBI-treated group.

We then investigated the potential role of *H. erinaceus* mycelia in reducing oxidative stress and enhancing antioxidant enzyme activity in an mTBI model. We also examined the involvement of the Nrf2 signaling pathway and the expression of phospho-CAMP-response element-binding (p-CREB) in this process. The CREB protein is known for its pro-survival effects on neuronal differentiation, survival, and neurite outgrowth and plays a role in microglial activation and the regulation of inflammation-related gene expression through NF-κB modulation [36,37]. Phosphorylation of CREB can induce the production of anti-inflammatory cytokines in activated microglia [38,39,40]. According to Figure 5, the p-CREB expression in both the cerebral cortex and subcortex decreased in the mTBI rats group. However, treatment with erinacine C and oral feeding of *H. erinaceus* mycelium induced p-CREB protein expression (Figure 5A,B). We performed immunohistochemical staining on the cerebral cortex and subcortex neurocytes to evaluate the antioxidative gene expression and protein expression related to protective mechanisms against neuronal injury in cerebral concussion. We assessed the expression of catalase (CAT), glutathione reductase (GSR), thioredoxin reductase (TrxR), and superoxide dismutase (SOD). There was a significant increase in protein expression of these antioxidant enzymes in the cortex and subcortex of rats receiving *H. erinaceus* mycelium or erinacine C injection compared to the untreated mTBI group (Figure 5, # < 0.05). Thus, *H. erinaceus* mycelium and erinacine C treatments may contribute to the reduction of oxidative stress and activation of antioxidant defense mechanisms in the brain, potentially the induction of p-CREB expression.

### 3.4. Immunohistochemistry Stain of NF-E2-Related Factor 2 Proteins on Brain Histopathology by H. erinaceus Mycelium and Erinacine C in Animal Model Rats with Mild Traumatic Brain Injury

**Figure 6:**
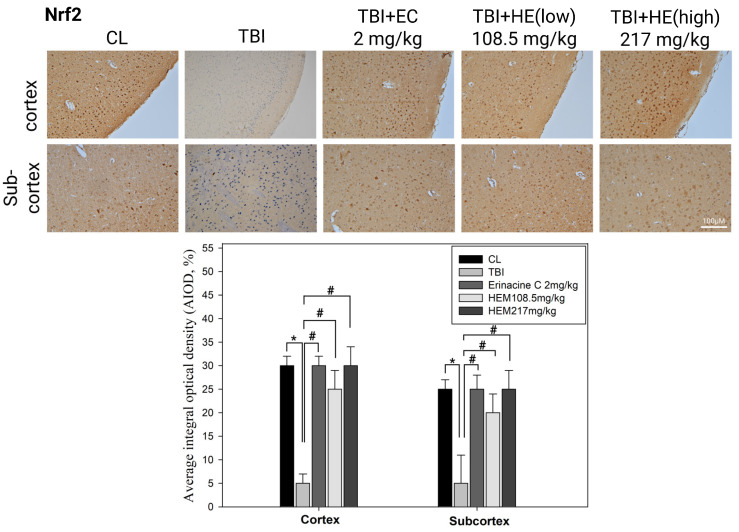
Effect of *Hericium erinaceus* mycelium (HEM) and its isolated erinacine C on brain histological NF-E2-related factor 2 (Nrf2) protein expression in the mild traumatic brain injury (mTBI) animal model. Rats with mTBI were treated without or with oral HEM administration (108.5, 217 mg/kg) and intraperitoneal erinacine C injection (2 mg/kg). Rats were sacrificed, and the brains were separated. The results show representative brain sections stained for the control group (CL), rats with mTBI, HEM, and its isolated erinacine C treatment staining of the brain tissue. Evaluations of cytosol protein expression were quantitative in the cortex and subcortex zones. The positive stained areas from three randomly selected observation fields were evaluated. Levels of proteins were quantitatively estimated through immunohistochemical analysis by average integrated optical density. Data are expressed as the mean ± standard deviation of independent experiments. *n* = 6. * *p* < 0.05, as compared to the control group; # *p* < 0.05, as compared to the mTBI-treated group.

Next, we investigated whether *H. erinaceus* mycelium has the ability to protect the brain against mTBI-induced brain injury in mice and whether this neuroprotective effect is mediated through modulation of the Nrf2 pathway [41]. The administration of *H. erinaceus* mycelium regimens significantly induced the expression of Nrf2 in the cortex and sub-cortex of the cerebral region compared to the untreated mTBI group (Figure 6, # < 0.05). Hence, *H. erinaceus* mycelium could exert neuroprotective effects against mTBI-induced brain injury by modulating the Nrf2 pathway and enhancing antioxidative responses in the brain.

### 3.5. Erinacine C Reduction of Lipopolysaccharide (LPS)-Induced Microglia Cells Activation in Primary Mixed-Glia Cultures

**Figure 7:**
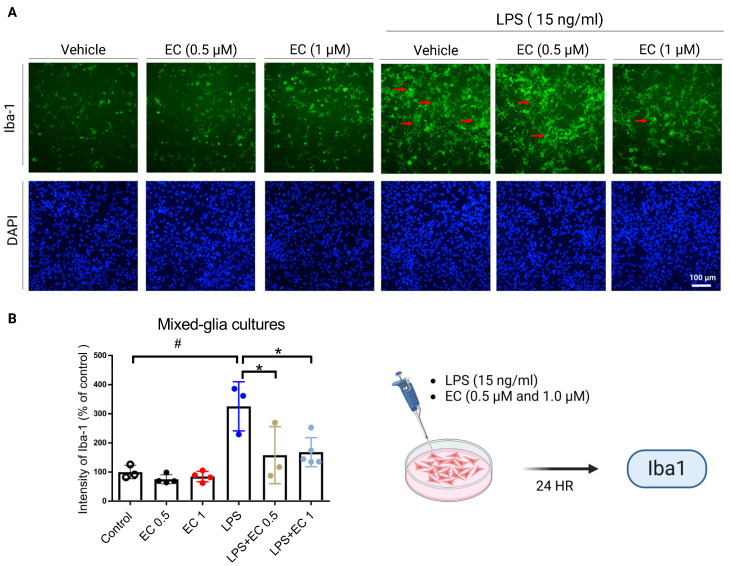
Erinacine C prevention of neuron inflammation in mixed glia culture cells with endotoxin (LPS). (**A**,**B**) Cell numbers of the microglia cells treated with or without LPS, or LPS and EC (0.5 μM and 1.0 μM) at 24 h measured by ionized calcium-binding adaptor molecule 1 (Iba1) staining and observation by fluorescence microscopy. The red arrows indicate the activated microglia cells with nuclei at a magnification of ×200. The cells from 10 fields were counted (200× magnification) of each sample. The statistical analysis results are presented as the means of cells and were calculated per microscope fields per group. # *p* < 0.05, as compared to the control group; * *p* < 0.05, as compared to the LPS-treated group.

**Figure 8:**
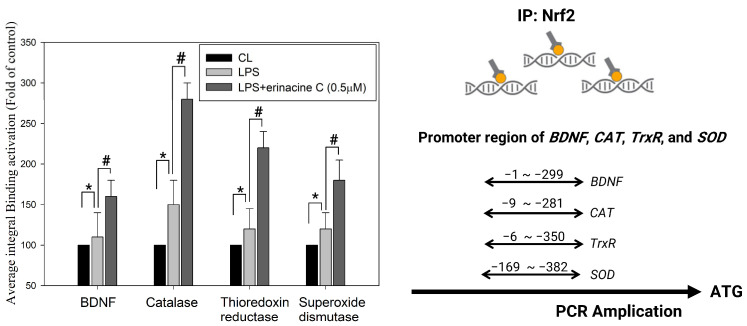
Chromatin immunoprecipitation-quantitative polymerase chain reaction (ChIP-qPCR) determination of binding of NF-E2-related factor 2 (Nrf2) to proximal promoters of genes altered in lipopolysaccharide (LPS)-induced mixed glia cells with erinacine C treatment. Erinacine C facilitated the binding of Nrf2 to the promoter regions of *BDNF*, *CAT*, *TrxR*, and *SOD* genes. The chromatin immunoprecipitation assay was performed using antibodies against Nrf2 and *BDNF*, *CAT*, *TrxR*, and *SOD* genes promoters (the target sites, as described in the Materials and Methods) in the precipitated DNA, which was amplified by qPCR using specific primer sets. BV2 cells were incubated with or without LPS and treated with erinacine C at various concentrations for 24 h. The effect of erinacine C treatment on specific genes was calculated by the ΔΔCt method. The quantitative data are presented as the mean ± standard deviation of three independent experiments in triplicate technical repeats. * *p* < 0.05, as compared to the control group. # *p* < 0.05, as compared to the LPS–treated group.

The effects of erinacine C, a derivative of *H. erinaceus*, on microglia activation and antioxidant enzyme expression were examined using reconstituted primary brain cell cultures, specifically mixed glial cultures containing microglia and astroglia [31]. LPS was used to induce microglia activation, which is characterized by the production of superoxide and inflammatory mediators [31]. LPS treatment increased the expression of Iba-1, a marker of activated microglia, in the mixed glial cultures at 6 h. However, the addition of erinacine C at concentrations of 0.5 and 1.0 µM significantly rescued the activated microglia, reducing their numbers by 44% and 46% compared to the LPS-treatment group, suggesting that erinacine C significantly affects the inhibition of microglia inflammation (Figure 7). To further explore the mechanisms underlying this effect, the researchers examined the regulation of antioxidant enzymes and the Nrf2 signaling pathway. Enzymatic antioxidants are important in protecting the brain from oxidative damage, which is implicated in neuronal degeneration and worsened response to mTBI [42]. We found that erinacine C treatment in the mixed glial cultures increased the binding of Nrf2 to the promoter regions of genes involved in antioxidant enzyme expression, including brain-derived neurotrophic factor (*BDNF*), *CAT*, *TrxR*, and *SOD*. ChIP assay results confirmed increased binding of Nrf2 to these gene promoter regions (Table 1). Furthermore, erinacine C treatment resulted in elevated levels of *BDNF*, *CAT*, and *TrxR* and enhanced *SOD* gene expression in the mixed glial cultures. Compared to the LPS-treated group, erinacine C increased the expression of these genes by 40%, 80%, 80%, and 45%, respectively (Figure 8), indicating that erinacine C could repress LPS-induced microglia activation through the inhibition of the Nrf2 pathway and induction of the expression of antioxidant enzymes, including *BDNF*, *CAT*, *TrxR*, and *SOD*. Hence, erinacine C may have potential neuroprotective effects by reducing inflammation and enhancing the cellular antioxidant capacity in the brain.

### 3.6. Erinacine C Decline of Lipopolysaccharide-Induced Mitochondrial Potential, Reactive Oxygen Species Production and Calcium Dysregulation in BV2 Microglia Cells

**Figure 9:**
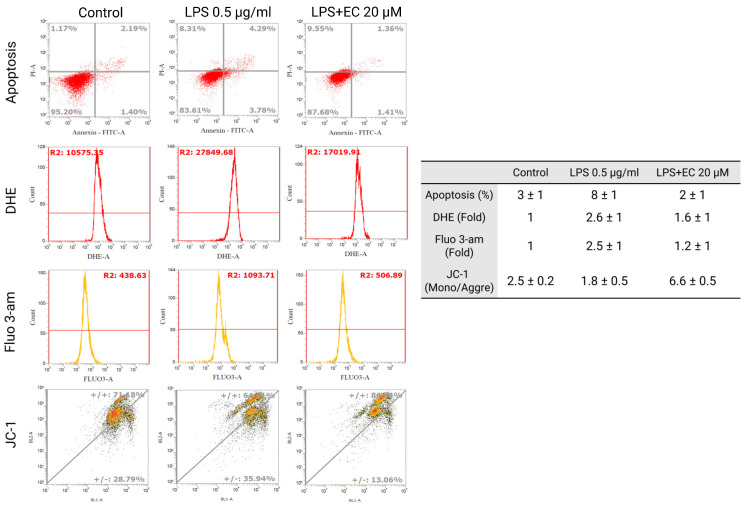
Erinacine C inhibited cell apoptosis and reactive oxygen species (ROS)/Ca^2+^ production and reverted the mitochondrial potential in BV2 cells. After erinacine C treatment, the BV2 cells were stained with annexin-V and propidium iodide, and the percentage of apoptotic cells was indicated after individual treatment. Intracellular ROS/Ca^2+^ levels in erinacine C-treated cells were quantified using FACS. The production of ROS/Ca^2+^ is presented as the fold change compared to the control group. Cells treated with erinacine C were subjected to staining with JC-1 for flow cytometry analysis. The ratio of red to green fluorescence intensity indicated potential-dependent accumulation in the mitochondria. Results are derived from three independent experiments and are expressed as mean ± standard deviation.

ROS, calcium signaling, and mitochondrial function have been implicated in microglial activation [15,16]. We further examined whether erinacine C could inhibit ROS and the influx of calcium production and mitochondria potential to suppress microglial activation and neuroinflammatory processes. Fluorescence-activated cell sorting analysis, which assesses the cell apoptosis with Annexin-V and PI staining, also revealed an obvious effect on apoptotic induction (3%, 8%, and 2%) in BV2 cells (Figure 9). The addition of erinacine C recovered LPS-induced apoptotic (annexin-V positive) cells. Moreover, the DHE (Dihydroethidium) assay as an indicator of ROS, showed that LPS treatment increased ROS (1 and 2.6) compared to that in the control group without erinacine C treatment, which was reversed by 1.6-fold after cotreatment with erinacine C. Flow cytometry experiments showed that erinacine C-treated BV2 cells exhibited reduced extracellular Ca^2+^ signaling by 1.2-fold, and presented polarization of the mitochondrial potential (DYm) marker JC-1 dye in terms of an increase in the aggregates red/monomers green fluorescence intensity ratio (2.5, 1.8, and 6.6) (Figure 9). Erinacine C exerted relatively high bioactivity and stalled LPS-induced BV2 cell death and ROS/Ca^2+^ generation, as well as depolarization of the mitochondrial potential of these cells.

## 4. Discussion

Commonly known as a concussion, mTBI can induce cellular and molecular alterations in the brain, causing neuronal damage [43]. Following mTBI, the brain experiences an immediate and exaggerated inflammatory response involving the activation of microglia, the resident immune cells [1,4]. Oxidative stress, characterized by an imbalance between ROS production and the antioxidant defense system, plays a pivotal role in mTBI-induced neuronal injury [16]. Activation of antioxidant defense pathways is protective in mitigating neuronal damage after mTBI, for which various drugs have been assessed in animal experiments and clinical trials [44,45]. However, the precise mechanisms underlying the neuroprotective effects of the components of *H. erinaceus* mushroom, particularly erinacine C, remain poorly understood. Erinacine A, a derivative from cultured mycelia of *H. erinaceus*, can prevent neuronal injury through the modulation of in vivo activity of inducible nitric oxide synthase, p38 mitogen-activated protein kinase, and C/EBP homologous protein. Treatment of an MPTP model of Parkinson’s disease with *H. erinaceus* and its ethanol extraction demonstrated preventive and therapeutic actions in restoring dopaminergic degeneration and ameliorating motor dysfunctions [26]. In this study, another composition of erinacine C in *H. erinaceus* was 3 mg/g (0.3%) (Figure 1). The beneficial effects of erinacine C were also investigated in a rat model of mTBI-induced neuronal injury and microglial activation, employing post-treatment regimens (Figure 2). Erinacine C post-treatment effectively modulated motor neuronal dysfunctions, as revealed by the beam walking test using an in vivo mTBI animal model and mouse primary mixed glia culture cells with LPS stimulation (Figure 3). Furthermore, treatment with *H. erinaceus* mycelium and erinacine C significantly reduced motor disorders, neuronal death, and microglia activation in the cortical and subcortical brain regions (Figure 3, Figure 4 and Figure 7). Additionally, several neuron cell death assays confirmed that erinacine C treatment for 24 h reduced LPS-induced cell damage, as indicated by decreased levels of intracellular ROS, extracellular Ca^2+^ signaling, and reduced polarization of mitochondrial potential (ΔΨm) in BV2 cells (Figure 9).

Oxidative damage resulting from ROS production is crucial for various neurodegenerative conditions, including mTBI [15]. In this study, effective chemopreventive strategies were developed using natural compounds from edible mushrooms to prevent neuronal death [16]. Oxidative stress triggered by mTBI can initiate a cascade of events resulting in neuritic/neuronal degeneration and synaptic disconnection. Excessive ROS production disrupts cellular functions, induces mitochondrial dysfunction, triggers inflammation, and leads to neuronal death. Enzymatic antioxidants can reduce ROS levels and oxidative stress by potentially suppressing these degenerative processes and protecting against further damage and microglial activation [39,40]. Investigation of the effects of enzymatic antioxidants in the context of mTBI can provide valuable insights into their therapeutic implications. Upregulating the Nrf2 pathway in a rat model of mTBI has shown promise in attenuating oxidative stress and promoting neurological recovery [46]. Activation of the Nrf2 pathway augments the expression of antioxidant enzymes and molecules such as SOD, CAT, and GPx. Following Nrf2 activation, reduced brain edema, improved motor coordination, and enhanced cognitive function have been reported [44]. Our histopathological and immunohistochemical assays (Figure 5) provide unique evidence suggesting that *H. erinaceus* and erinacine C regulate endogenous antioxidant defense mechanisms, including *CAT*, *GSR*, *TrxR*, and *SOD*, that scavenge ROS and protect neurons from oxidative damage, thus holding potential benefits for mTBI. Our results (Figure 3 and Figure 6) further demonstrate that erinacine C inhibits brain edema, improves motor coordination, and enhances cognitive function through Nrf2 expression, as indicated by the beam walking test. These improvements likely result from the combined effects of reduction in oxidative stress, anti-inflammatory action, and protection of the mitochondria. Furthermore, treatment of primary mixed-glia cultures with erinacine C increased the binding of Nrf2 to the promoter regions of genes such as *BDNF*, *CAT*, *TrxR*, and *SOD1*, as determined by the ChIP assay (Figure 8). Enzymatic and non-enzymatic antioxidants protect the brain against oxidative damage and are major defense enzymes against superoxide radicals. Activation of Nrf2 improves cellular antioxidant capacity and protects neurons from oxidative stress-induced damage, suggesting this as a possible mechanism for the upregulation of BDNF following LPS stimulation [47,48,49].

Brain injuries, such as mTBI and other neurodegenerative diseases, are often accompanied by oxidative stress, impaired energy homeostasis, neuroinflammation, and apoptosis, leading to compromised function of the brain cells [49]. The effects of the cortex and subcortex regions for analysis in mTBI animal models result from higher cognitive function and metabolic demand, making them more sensitive to oxidative stress-induced damage following injury under complex neuronal networks. Expectations from other brain regions might also exhibit specific responses to mTBI, including the brainstem, cerebellum, basal ganglia, and white matter. These brain regions also play crucial roles and might exhibit distinct responses or alterations following mTBI, providing a comprehensive understanding of the injury’s impact on various brain functions, such as basic physiological indications, motor coordination, and myelin integrity or axonal damage. Our study revealed that mTBI induced motor neuronal dysfunctions, as evidenced by the beam walking test. Additionally, the cortex, subcortex, and hippocampus regions of the brain were particularly susceptible to oxidative stress (Figure 3, Figure 4, Figure 5 and Figure 6). These results demonstrated that *H. erinaceus* mycelium and erinacine C possess properties that can protect the brain against neuronal injury, and microglial activation in mTBI rat models should be listed as limitations of the study of female rats. Behavior analysis should be performed through a Morris water maze test, as well as Y-maze and beam-walking tests. However, administration of H. erinaceus and erinacine C in rat models of mTBI showed improvement in memory deficits, and further validation of these findings is still required by the Morris Water Maze test and Y-Maze test [50]. The p-CREB pathway activation has been associated with enhanced expression of neuroprotective genes, promotion of cell survival pathways, inhibition of apoptosis, modulation of oxidative stress, anti-inflammatory effects, and synaptic plasticity, and is crucial in learning, memory, and motor processes [50]. Our data demonstrated a significant increase in the level of p-CREB in the cortex and subcortex structures of the mTBI brain following treatment with *H. erinaceus* mycelium and its ethanol extraction of erinacine C (Figure 5).

Natural phytochemicals and dietary foods improve motor and nonmotor symptoms, delay disease progression, and exhibit neuroprotective effects in experimental models, including the rat model of mTBI [51]. Compounds such as curcumin, resveratrol, epigallocatechin-3-gallate, and flavonols, such as epicatechins, have been shown to cross the blood-brain barrier in rodents following oral or intravenous administration [47,51,52]. Besides erinacine A and erinacine S, *H. erinaceus* mycelium extracts contain other erinacine C compounds that have exhibited brain-protective effects in the treatment of neurodegenerative disorders. In addition to a rat model of the mTBI model, two other inflammation-based progressive neurodegenerative models include a moderate controlled cortical impact (CCI) model in C57BL/6 mice and repetitive mTBI models [17,46,53]. Before initiating clinical trials, the neuroprotective effects of erinacine C in these oxidative stress-based progressive neuronal injury models must be assessed. Further studies are also required to examine whether erinacine C targets multiple signaling pathways and elucidate potential crosstalk mechanisms. Overall, our findings suggest that erinacine C holds promise as a natural candidate for the mTBI treatment. Although Erinacine C has shown potent neuroprotective effects in the mTBI model, we also plan to apply synthetic erinacine C into the modified weight-drop technique-based progressive mTBI models before the entrance of the clinical trial. *H. erinaceus* mycelium extracts may contain a combination of bioactive compounds, and we might have aimed to investigate the potential synergistic effects of different components present in the extracts. However, working with erinacine C alone might overlook potential interactions or synergies with other compounds present in the mycelium extract, which could contribute to the observed effects. This study could provide a more comprehensive understanding of the therapeutic potential of all extracts. The use of different doses of *H. erinaceus* mycelium extracts in a study is often aimed at exploring dose-dependent effects and determining an optimal or effective dosage. Without the specific details of the study before, here are some potential reasons for using both high and low amounts of *H. erinaceus* mycelium extracts, along with suggestions for treatment in this study, such as safety and the robustness of mechanisms of action.

Immunohistochemistry (IHC) is a valuable technique for visualizing the spatial distribution of several antioxidant enzymes and phospho-CREB levels within brain tissues, but it has limitations when it comes to providing quantitative data. From a future perspective, to further validate and quantify changes of indicated proteins, incorporating additional methods such as Western blotting (WB) or mRNA analysis is crucial. These techniques can offer more quantitative insights into the molecular changes induced by treatments of *H. erinaceus* mycelia and erinacine C in an mTBI model. We do need a more comprehensive exploration of pro- and anti-inflammatory markers beyond Iba-1, a marker for microglial activity. They are considering conducting a new experiment to address these concerns, such as the selection of Pro-inflammatory markers (e.g., TNF-α, IL-1β, IL-6) and anti-inflammatory markers (e.g., IL-10, TGF-β) relevant to the study, following an appropriate primary mixed-glia cultures ex vivo that responds to LPS treatment and allows for the assessment of both pro- and anti-inflammatory markers. In future directions, the effects of erinacine C, a derivative of H. erinaceus, on astrocyte activity as well as Pro- and anti-inflammatory markers expression are determined to assess changes in the expression of specific proteins associated with inflammation by ELISA or qPCR to quantify these markers in cell culture supernatants or lysates.

## 5. Conclusions

**Figure 10:**
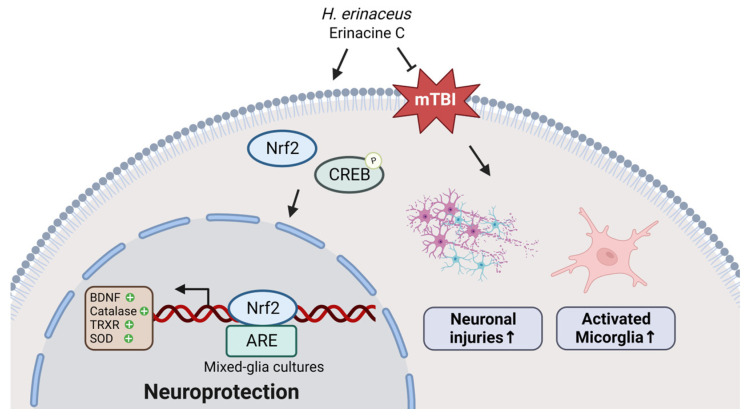
Schematic presentation of the molecular mechanism of the action of *Hericium erinaceus* mycelium (HEM) and erinacine C on the protection against neuronal injury and microglia activation in rat models of mild traumatic brain injury (mTBI) through the Nrf2 pathway. In the nucleus, Nrf2 promotes transcriptional activation of antioxidants enzymes (brain-derived neurotrophic factor (*BDNF*), catalase (*CAT*), thioredoxin reductase (*TrxR*), and superoxide dismutase (*SOD*) by erinacine C by binding to the promoter regions of the downstream target genes, thereby triggering the antioxidant defense system and suppressing microglial activation in primary mixed-glia cultures. HEM and its components erinacine C in the treatment of mild brain traumatic injury (mTBI)-induced neuronal damage and microglia activation that could protect from neuronal injury.

In conclusion, our study demonstrates that *H. erinaceus* mycelium and erinacine C possess properties that can protect the brain against neuronal injury and microglial activation in mTBI rat models. We elucidated the specific mechanisms underlying these effects, which involve the activation of the Nrf2 pathway. Further, erinacine C treatment of primary mixed-glia cultures promoted the binding of Nrf2 to the promoter regions of *BDNF*, *CAT*, *TrxR*, and *SOD1* genes, resulting in the activation of the antioxidant defense system and the inhibition of microglial activation. These findings suggest the molecular mechanisms by which *H. erinaceus* exerts its beneficial effects and highlight the role of antioxidant defense pathways in mitigating neuronal injury and microglial activation, thereby reducing brain damage in cases of mTBI (Figure 10).

## Figures and Tables

**Table 1:** The specific primers.

BDNF −1 to −299 bp NCBI Reference Sequence: NM_001285421.1 Sequence ID: NC_000068.8
5′-TTGTTTTGTGCCGTTTACCA-3
5′-GGTAAGAGAGCCAGCCACTG-3
Catalsase −9 to −281 bp NCBI Reference Sequence: NM_009804 Sequence ID: NC_000068.7
5′-ATTGGACCCTGAGCTGTGAC-3′
5′-GGAGAAGGCAATCTTGTTGG-3′
Thioredoxin reductas −6 to −350 bp NCBI Reference Sequence: NM_015762 Sequence ID: NC_000076.7
5′-CGATTCTGGTTCCCAACATT-3′
5′-TAAAGAGCTGCGGGTTCCTA-3′
Superoxidase dismutase −169 to −382 bp NCBI Reference Sequence: NM_011434 Sequence ID: NC_000082.7
5′-ATCTTGGCGCATCTCAACTT-3′
5′-GTGCGGACTGAGAAAGTTCC-3′

## Data Availability

All relevant data are included in the paper.
